# Supplementary material for: Conditional chemoconnectomics (cCCTomics) as a strategy for efficient and conditional targeting of chemical transmission
Source: eLife. 2024 Apr 30;12:RP91927. doi: 10.7554/eLife.91927 (PMC11060718; doi:10.7554/eLife.91927)
Supplement: Figure 3—source data 3. [file elife-91927-fig3-data3.zip › Figure 3 source data3 part 1.zip!/Figure 3-Source data 3-part1/1081-1-CRISPResso_Report_8zosk6yj.zip!/Nucleotide_percentage_quilt.pdf]

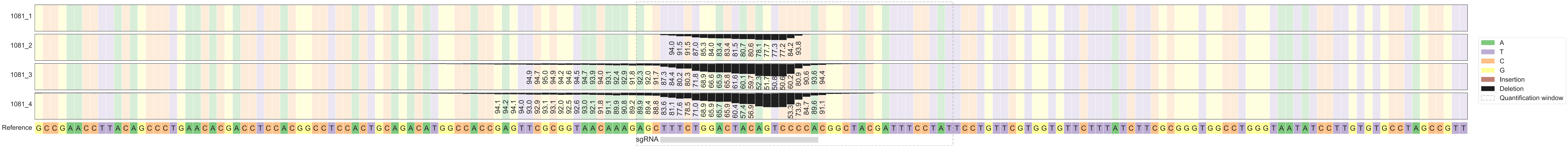
1081_1
1081_2
1081_3
1081_4
Reference
GCCGAACCTTACAGCCCTGAACACGACCTCCACGGCCTCCACTGCAGACATGGCCACCGAGTTCGCGGTAACAAAGAGCTTTCTGGACTACAGTCCCCACGGCTACGATTTCCTATTCCTGTTCGTGGTGTTCTTTATCTTCGCGGGTGGCCTGGGTAATATCCTTGTGTGCCTAGCCGTT
sgRNA
A
T
C
G
Insertion
Deletion
Quantification window
